# Feasibility and performance of eye movement perimetry for glaucoma triage in Tanzania: A prospective cross-sectional study in a real-world setting

**DOI:** 10.1371/journal.pgph.0007237

**Published:** 2026-09-25

**Authors:** Arun J. Thirunavukarasu, Victor H. Hu, Pete R. Jones, Elliott H. Taylor, Einoti Matayan, Sia Mbishi, Edith Macha, Tara Mtuy, David P. Crabb, William Makupa, Matthew J. Burton, Heiko Philippin

**Affiliations:** 1 International Centre for Eye Health, London School of Hygiene & Tropical Medicine, London, United Kingdom; 2 School of Health and Medical Sciences, City St George’s, University of London, London, United Kingdom; 3 Institute of Ophthalmology, University College London, London, United Kingdom; 4 Kilimanjaro Christian Medical Centre, Moshi, Tanzania; 5 Eye Center, Medical Center, Faculty of Medicine, University of Freiburg, Freiburg, Germany; 6 Christian Blind Mission, Bensheim, Germany; PLOS: Public Library of Science, UNITED STATES OF AMERICA

## Abstract

Triaging access to glaucoma diagnostics could allow patients to receive earlier care to slow disease progression. There is a particular need in lower-resource settings where the cost and lack of portability of standard automated perimetry (SAP) restrict access. Eye movement perimetry (EMP) uses eye-tracking technology to provide a rapid assessment of the visual field using a tablet computer. A cross-sectional study in Tanzania was undertaken to assess the feasibility of EMP as an initial glaucoma triage tool. EMP hit rate correlated with SAP mean deviation in 370 eyes (Spearman’s rank correlation coefficient, ρ = 0.441, *p* < 0.001) and graphical comparisons indicated good localisation of defects. In emulated triage of 232 participants, EMP exhibited very high (100.0%) sensitivity (95%CI: 95.5 to 100.0%) in identifying eyes with mean deviation ≤ -6 dB), meaning that a high EMP hit rate was strong evidence of a healthy visual field. However, specificity was 20.7% (95%CI: 15.0 to 27.8%), meaning that many individuals with no severe field defect would require SAP to confirm this. Specificity was limited by EMP test failure, which occurred in 75 of 480 eyes (15.6%), as well as export or processing failure which occurred in 35 eyes (7.3%). Participants preferred EMP to SAP testing and agreed that it was easy to perform. In a real-world setting, EMP rapidly and accurately identifies patients without a significant visual field defect. However, refinement is required to reduce the high number of unnecessary referrals or false positives for EMP to prove useful as a triage tool.

## Introduction

Glaucoma is a group of progressive optic neuropathies that collectively represents the most common cause of irreversible blindness worldwide [1,2]. Glaucomatous blindness is more common in lower-income areas, such as parts of sub-Saharan Africa, due to limited access to diagnosis and intraocular pressure-lowering treatments [3]. In addition, the predominant phenotype of primary open-angle glaucoma in sub-Saharan African individuals tends to progress faster than in Caucasian individuals, narrowing the temporal window within which intervention can prevent blindness [3–5]. Detecting and diagnosing glaucoma is challenging, generally requiring multiple investigations that depend on expensive equipment, trained operators, and time for repeated tests to monitor changes [4].

The central investigation in identifying and staging glaucoma is perimetry: machine-based assessment of patients’ visual fields [6,7]. Standard automated perimetry (SAP) is the reference standard, often performed with the Humphrey Field Analyser, Octopus Perimeter, Medmont Automated Perimeter, or Henson Perimeter [8]. However, SAP is time-consuming, requires a trained operator, and is not enjoyable for patients because of its duration and requirement for focus. SAP machines are too large to transport and expensive to purchase and maintain, limiting their availability in lower-income settings. Because of this lack of access to diagnostics (in addition to therapeutics), glaucoma is a significant public health problem in sub-Saharan Africa [3,9,10]. A large proportion of patients exhibit late-stage disease with significant visual impairment on initial presentation, which cannot be reversed [11,12]. In Tanzania, up to 98.5% of rural residents with glaucoma are unaware that they have the disease, likely due to having never consulted an eye care professional [13].

Eye movement perimetry (EMP) employs an inexpensive eye tracking device with a portable tablet or laptop computer for an alternative to SAP [14–17]. Its output score (EMP ‘hit rate’) is calculated based on participants’ propensity to fixate on peripheral stimuli when presented on the screen. In early feasibility studies, the EMP hit rate correlated with SAP mean deviation and localised scotomas apparent on SAP results with good specificity, indicating potential clinical utility [15,18]. Moreover, EMP is much faster to complete than SAP as there are fewer stimuli per test, and EMP has also been better tolerated than SAP by patients in the UK [15].

An effective triage system with lower cost, better ease of use, and greater portability than SAP systems could reduce the burden of patients travelling to access glaucoma diagnostics in lower-income countries. This could in turn allow patients to receive earlier pressure-lowering therapy and thereby reduce disease progression. However, it is crucial that a proposed triage test does not provide false reassurance for patients with a visual field defect—*i.e.,* that the triage test exhibits high sensitivity. Specificity is of secondary importance: higher performance suggests that a greater proportion of individuals with no visual field defect would be correctly excluded, but lower performance may be acceptable as patients with a referrable result would merely receive further non-invasive investigations. However, lower specificity may lead to problems of overburdening limited capacity for investigations and could incur unnecessary costs of travel, inconvenience, and worry to individuals without a visual field defect.

This study aimed to establish the feasibility of EMP for triage of individuals with possible glaucoma in a low-resource setting (Moshi, Tanzania). Four aspects of feasibility were considered: test failure rate and reasons for EMP not being completed, eye-level performance of EMP predicting visual field defects on subsequent SAP, person-level triage performance of EMP and the theoretical reduction in demand for SAP, and the acceptability of EMP in comparison to SAP for participants.

## Methods

### Ethics statement

This study adhered to the tenets of the Declaration of Helsinki. It was approved by the Tanzanian National Institute of Medical Research Ethics Committee (NIMR/HQ/R.8a/Vol.IX/3669), Tanzania Medicines and Medical Devices Authority (TMDA-WEB021/CTR/0013/02), Kilimanjaro Christian Medical Centre Ethics Committee (2481), and the London School of Hygiene & Tropical Medicine Ethics Committee (17824). The study was explained to potential participants in Kiswahili or English, and written informed consent was obtained before enrolment.

The open source EMP test used in this study (previously but no longer referred to as ‘Eyecatcher’) is a tablet computer application that works with a clip-on eye-tracking device to facilitate rapid and portable visual field assessment [19]. Unlike SAP, subjects are free to move their eyes around, do not have to press a button to signal light perception, and are not required to undergo any head restraint [15]. Participants are challenged to look at light stimuli as they appear on the tablet computer screen, with eye-tracking used to identify the start and end fixation points for saccades to deduce whether stimuli were seen or unseen. Small scale studies suggest that the EMP hit rate correlates with SAP mean deviation, and may reliably separate glaucoma patients from healthy controls [15,18]. In this study, EMP was used as a research tool and not developed as a commercial device. Its MATLAB source code is available upon request.

Here, a prospective cross-sectional study in real-world conditions was undertaken in Tanzania to explore the potential utility of the EMP test as a glaucoma triage tool in lower-income settings where access to SAP is limited. The prospectively registered protocol is available in S1 Text. Between March 2022 and December 2023, consecutive participants were recruited from ophthalmology clinics in Kilimanjaro Christian Medical Centre, Moshi, Tanzania, with purposefully broad inclusion criteria to capture the heterogeneous population upon which a glaucoma triage tool might be deployed. Specifically, participants were recruited if they met the following criteria: aged 18 years or older, visual acuity of 6/24 Snellen or better in the eye(s) to be included in the study, able to understand and engage with perimetry tests, and having provided written informed consent. Participants were not permitted to enrol in the study more than once.

**Fig 1:**
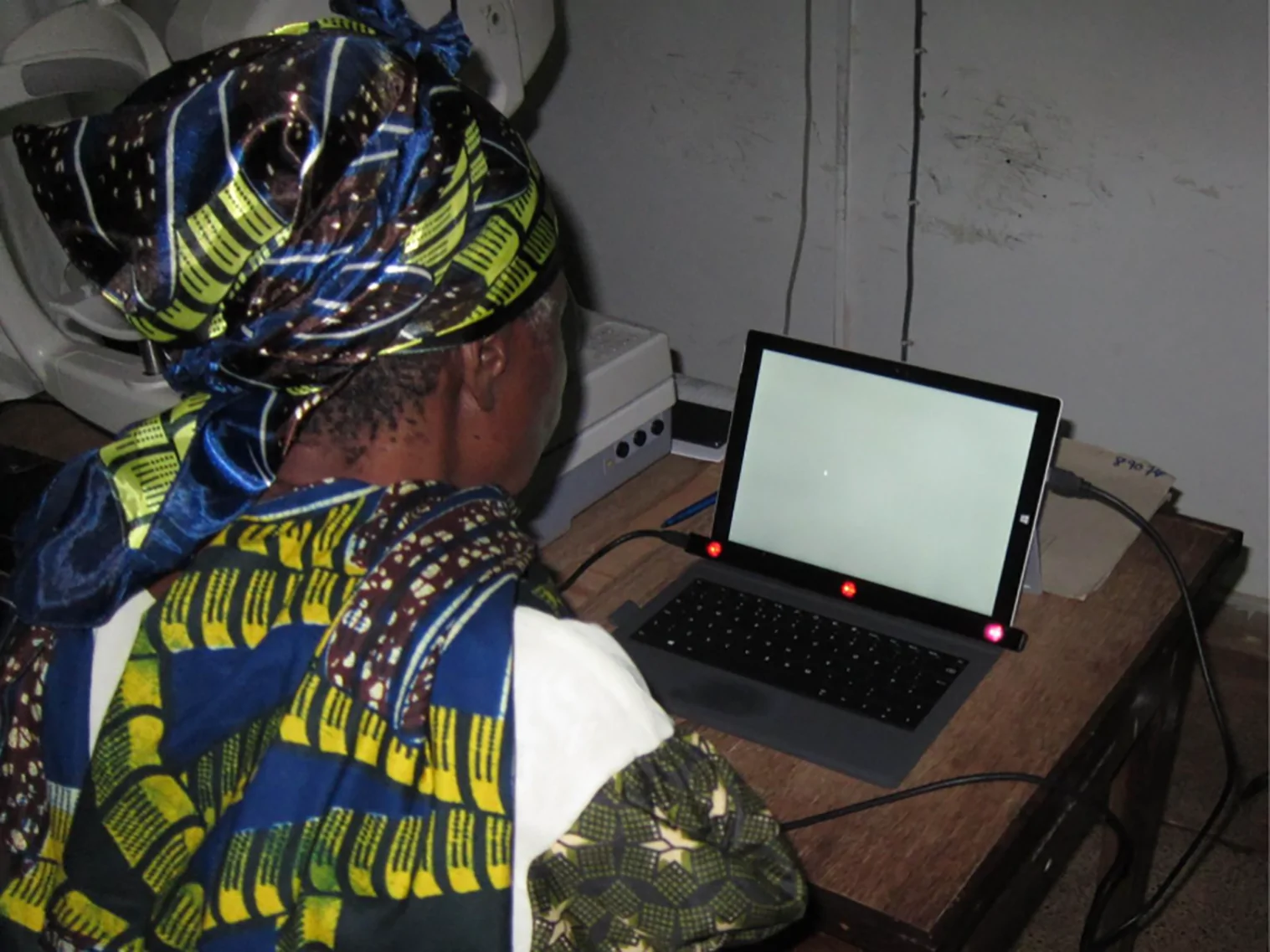
Study participant completing the eye movement perimetry (EMP) test at Kilimanjaro Christian Medical Centre in Moshi, Tanzania. A light stimulus can be seen left of centre on the screen of the tablet computer, of Goldmann size III, and 6 dB brighter than a normative threshold for an age-similar, normally sighted adult population undertaking standard automated perimetry (SAP). The eye tracking device is fixed just underneath the screen of the tablet computer. To complete the test, participants are tasked with looking at light stimuli on the screen as they appear. The EMP hit rate is calculated based on the proportion of points that the participant fixated at each retinal location. *Courtesy of Heiko Philippin.*

During their clinic attendance, participants were asked to complete the EMP test using a tablet computer (Surface Pro 7, Microsoft Corporation, Redmond, Washington, US) with an EyeX (Tobii, Stockholm, Sweden) eye-tracking device attached to the front of the computer (Fig 1). The eye-tracking data were used in near-real-time to determine where the participant was fixating. The test involved a brief initial nine-point calibration procedure requiring participants to follow supra-threshold light stimuli. Participants who successfully completed calibration progressed to the full EMP test with Goldmann Size III stimuli of fixed differential luminance 6 dB more intense than normative thresholds for an age-similar normally-sighted adult performing SAP [18]. A research assistant was available to administer the test and troubleshoot; as well as to record if the test was not completed and document any specific reasons. SAP was performed within three months (often on the same day) using the Swedish Interactive Thresholding Algorithm (SITA) 24–2 programme on the Humphrey Visual Field HFA II 740i visual field analyser (Carl Zeiss Meditec AG, Jena, Germany).

Participants were also surveyed to gauge their opinion of the EMP test relative to SAP and explore the acceptability of the new test (S1 Text, Appendix B). Participants were asked whether they felt the EMP test was easy to use, whether EMP was easier than SAP, and whether SAP was easier than EMP. Participants responded through Likert scales with answers of ‘strongly disagree’, ‘disagree’, ‘neither agree nor disagree’, ‘agree’, or ‘strongly agree’. In addition, participants were asked to provide any other comments or feedback on either or both of the visual field tests, which were recorded as free text.

A 70% EMP hit rate was used as the proposed threshold for onward diagnostics and this was defined prospectively [18]. The reference standard used to define a visual field defect was mean deviation on SAP, with mean deviation less than -6 decibels (dB) used as the definition of a moderate to severe visual field defect [20]. At eye-level, the paired EMP hit rates and SAP mean deviations were plotted graphically to provide an overview of their distribution and indicate how changing referral thresholds would affect diagnostic statistics. Receiver operating characteristic (ROC) curves were also produced to quantify the overall ability of EMP to discriminate between healthy and suspicious visual fields [21]. To measure correlation between EMP hit rate and SAP mean deviation, Spearman’s rank correlation coefficient (ρ) was calculated. Individual global plots from EMP and SAP results were compared to observe whether the new test captured the location of field defects effectively. In addition, a person-level triage emulation was undertaken where a single eye failing to complete EMP or meeting the 70% threshold was sufficient for further investigation; allowing for calculation of the proportion of individuals with visual field defects that would be correctly identified (sensitivity), and of the proportion of individuals with no visual field defect that might be discharged (specificity). Therefore, while eyes with missing data were excluded from the eye-level analyses of diagnostic accuracy, they were retained in the person-level analysis of triage performance to maximise realism.

Test failures and reasons for failure were analysed graphically and qualitatively. Participant feedback was graphed to illustrate the extent of agreement and disagreement with statements about the EMP test’s ease of use relative to SAP. Failure cases were categorised and tabulated to compare their frequency. Deductive thematic analysis was undertaken by two masked researchers to categorise participant comments to explore the valence and subject of participants’ opinions. Valence (positive, negative, neutral, or unclear with respect to EMP and/or SAP) and subject categories were determined by an initial discussion after each researcher appraised the results. Disagreements in valence or subject judgements were resolved by subsequent discussion.

A formal power calculation was not performed as the study was designed to assess feasibility; it was anticipated that approximately 250 participants would be recruited in line with the project timeline and available support, and this was considered adequate to undertake a meaningful analysis. Results were analysed in Stata (StataCorp LLC, College Station, Texas, USA) and R (R Foundation for Statistical Computing, Vienna, Austria). Where possible, all statistics were reported with corresponding 95% confidence intervals (95%CI). Visualisations were produced in Affinity Designer 2 (Serif Ltd, West Bridgford, United Kingdom). The study data are available for others to use for academic or research purposes [22].

## Results

### Participant demographics and test failure rate

480 eyes of 248 individuals were enrolled. The median age on the day of testing was 56.5 years (range 19.1 to 86.5 years), there were around twice as many female participants as male participants, and the most common tribe represented was the Chagga. Of the 480 eyes, 370 eyes (77.1%) from 208 participants (83.9%) undertook both the EMP and SAP tests and had data exported for analysis. Of these, 99 eyes (21%) were glaucomatous with a confirmed diagnosis, 137 eyes (29%) were glaucoma suspects, and 244 eyes (51%) had no glaucoma. 163 eyes (34%) exhibited a visual field defect (mean deviation on SAP ≤ -6 dB). The distributions of age, sex, tribe, and field defect status was similar in the subset of participants who did and did not complete the EMP test (Table 1; *p* > 0.05).

**Table 1:** Demographics of the recruited cohort as well as the subset of individuals that completed the EMP test. There was no association between likelihood of completing the EMP test and age, sex, tribe, or visual field status (*p* > 0.05). EMP = eye movement perimetry, OR = odds ratio, 95%CI = 95% confidence intervals.

Characteristic	All	Completed the EMP test	Did not complete the EMP test	Comparison between participants or their weaker eye that did and did not complete the EMP test
**Number enrolled**				
Participants	248	209	39	
Eyes	480	405	75	
**Age**				*t*-test, *p* = 0.638
Mean (standard deviation)	53.5 (16.3) years	53.3 (16.4) years	54.6 (15.6) years	
Range	19.1 to 86.5 years	19.1 to 86.5 years	19.3 to 81.4 years	
**Sex**				OR 1.16 (95%CI: 0.53 to 2.48); *p* = 0.717 (Fisher’s exact test)
Females	312	265	47	
Males	168	140	28	
**Ethnic groups**				*p* = 0.019 (Fisher’s exact test simulated over 2,000 permutations)
Chagga	137	117	20	
Pare	29	24	5	
Iraqw	7	6	1	
Sukuma	6	5	1	
Other	69	57	12	
**Mean deviation**				
≤ -6 dB (marked field defect)	104	85	19	OR 1.38 (95%CI: 0.66 to 2.92); *p* = 0.380 (Fisher’s exact test)
> -6 dB (no marked field defect)	144	124	20	
≤ -2 dB (mild field defect)	221	188	33	OR 0.62 (95%CI: 0.22 to 2.01); *p* = 0.398 (Fisher’s exact test)
> -2 dB (no mild field defect)	27	21	6	

22 eyes (4.6%) of 19 participants (7.7%) did not complete SAP testing. 75 eyes (15.6%) of 39 participants (15.8%) did not complete EMP testing. In addition, EMP results data export or processing failed after 35 completed tests (7.3%). Frequently, there were multifaceted reasons for EMP test failure, but the most common factors were difficulties with the initial calibration task, and anomalous eye tracking data such as high variability in estimated distance between head and device (Table 2). Other contributors to test failure included hardware or software issues, ocular surface disease, or limited ability to fixate and follow the stimulus (Table 2). The sole hardware issue was a cable between devices not connecting, which was reported just once. Software failures were of eye-tracking (n = 2) and of initial calibration (n = 50) which may have been for a variety of underlying patient-centric or system-centric reasons. Other eye issues included pain (n = 2), epiphora (n = 2), and ocular disease (n = 3).

**Table 2:** Reasons for EMP test failure documented by operators during the study. Initial calibration and anomalous eye tracking data were by far the most frequent cause of test failure. These likely reflect participants’ difficulty engaging with the test. Other issues were also mostly patient-centred, *e.g.*, inability to fixate, or ocular surface characteristics.

Reason for test failure	n
Anomalous eye tracking data	49
Calibration not working	50
Cooperativeness	2
Epiphora	2
Eye-tracking failure	2
Hardware issue	1
Inability to fixate	9
Lack of understanding	2
Ocular disease	3
Pain	2
Unknown (not documented)	2

### EMP correlation with conventional perimetry and prediction of visual field defects

**Fig 2:**
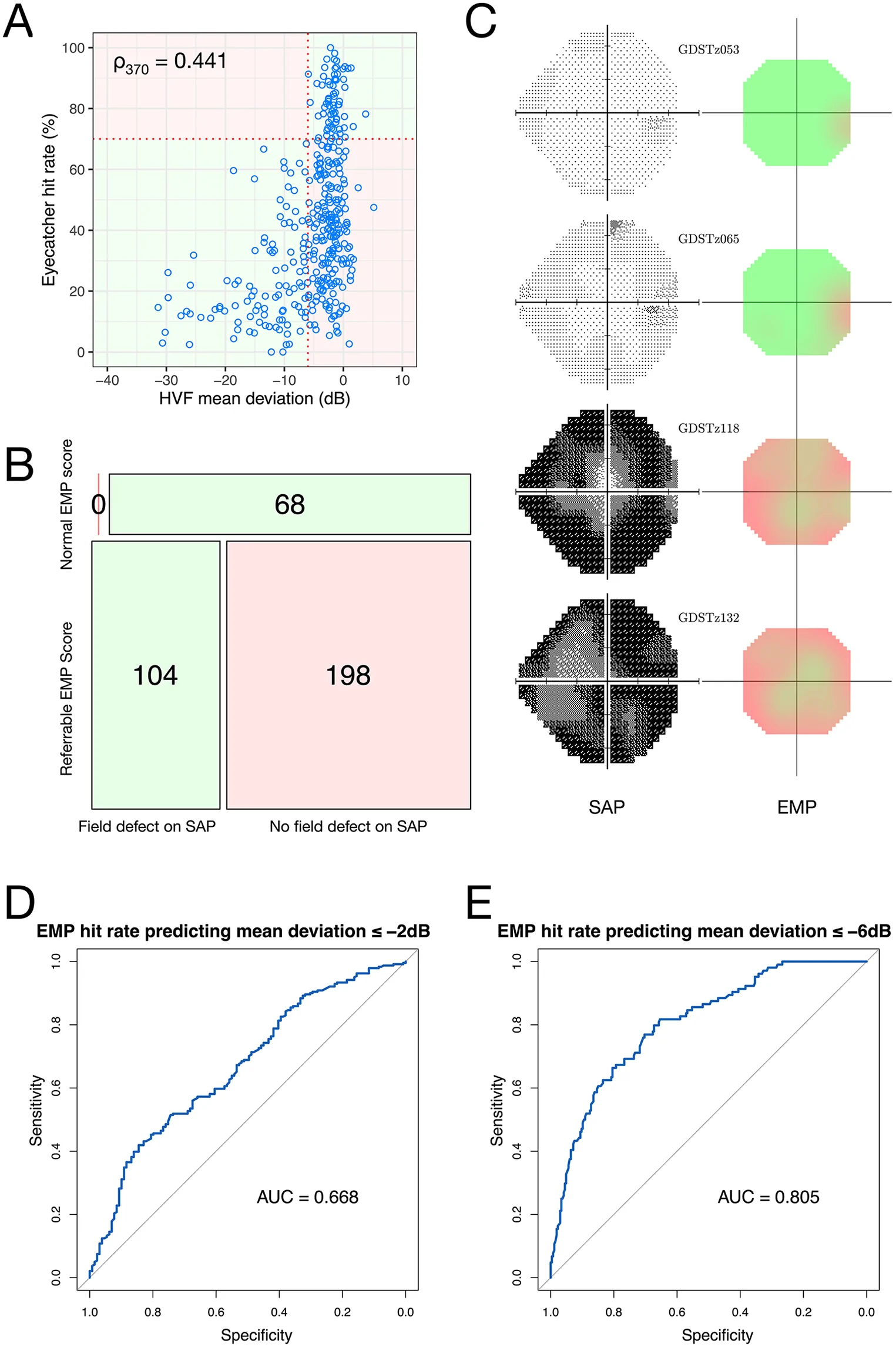
Performance of the eye movement perimetry (EMP) test in comparison with standard automated perimetry through Humphrey Visual Field standard automated perimetry (SAP) testing. **A)** Mean deviation on SAP results against EMP test performance; with a threshold EMP hit rate of 70%, all individuals exhibiting a visual field defect (defined as mean deviation < -6 dB) were correctly identified. There was statistically significant correlation between EMP hit rate and mean deviation on SAP (ρ = 0.44, *p* < 0.001). **B)** Mosaic plot depicting the diagnostic performance of the EMP test with a threshold for referral of 70%, and SAP mean deviation used as the reference standard. The shaded quadrants correspond to those of Fig 2A. The EMP test exhibited perfect (100%) sensitivity, and a specificity of 26%. **C)** Exemplar SAP results plotted alongside corresponding plots from EMP tests, showing the novel test captures field defects that are apparent on SAP. **D)** Receiver operating characteristic (ROC) curve for the performance of EMP predicting a mean deviation on SAP of -2 dB or lower (worse). Area under the curve (AUCROC) was 0.668 (95%CI: 0.609 to 0.728). **E)** ROC curve for the performance of EMP predicting a mean deviation of -6 dB or lower. AUCROC was considerably higher than for predicting more mild visual field defects: 0.805 (95%CI: 0.757 to 0.853).

Of 370 eyes that completed EMP and SAP tests, 302 EMP results were below the 70% hit rate threshold, suggesting possible visual field loss (Fig 2A). Of these, 104 eyes (34.4%) exhibited visual field defects on subsequent SAP testing, while 198 eyes (65.6%) exhibited no visual field defect on SAP testing. Of the 68 tests where EMP performance was deemed within normal limits, all eyes (100%) exhibited no visual field defect on SAP testing. There were no eyes that exhibited a good EMP hit rate but a visual field defect on SAP testing. These results are summarised in Fig 2B: 70% threshold sensitivity was 100.0% (95%CI: 96.4 to 100.0%), while specificity was 25.6% (95%CI: 20.7 to 31.1%). The area under the receiver operating characteristic curve (AUCROC) was higher with EMP predicting mean deviation of -6 dB or lower on SAP (AUCROC = 0.805, 95%CI: 0.757 to 0.853, Fig 2E) than in predicting mean deviation of -2 dB or lower (AUCROC = 0.668, 95%CI: 0.609 to 0.728, Fig 2D).

Graphical comparison of SAP and EMP result plots showed that the EMP test frequently mapped visual field defects similarly to standard automated perimetry (Fig 2C). However, the EMP test also indicated visual field defects in many participants with full fields and early defects (mean deviation > -6 dB) on SAP results (Fig 2B). Overall, the mean deviation in SAP results correlated with EMP hit rate (Spearman’s rank correlation coefficient, ρ = 0.441, *p* < 0.001), indicating that the global output of the EMP test reflected that of standard automated perimetry (Fig 2A).

### Person-level analysis of EMP triage performance

Of 232 individuals with SAP data available, 201 (86.6%) exhibited test failure or at least one EMP hit rate equal or lesser to 70%, which were the proposed triage criteria for further investigation of a potential visual field defect. This was most commonly due to both eyes scoring 70% or less on EMP (n = 127, 54.7%), rather than a single eye scoring 70% or less (n = 34, 14.7%), or test failure in a single (n = 16, 6.9%) or both (n = 9, 3.9%) eyes. Test failure in one eye with the contralateral eye meeting the EMP threshold occurred in 15 (6.5%) individuals.

All individuals with a visual field defect defined as mean deviation of -6 dB or worse were correctly identified in the triage emulation: sensitivity was therefore 100.0% (95%CI: 95.5 to 100.0%). However, specificity was just 20.7% (95%CI: 15.0 to 27.8%), indicating the proportion of individuals with no visual field defect that would have been correctly discharged and spared further investigation. The positive predictive value (PPV) of the emulated triage application was 40.8% (95%CI: 34.2 to 47.7%) and the negative predictive value (NPV) was 100.0% (95%CI: 89.0 to 100.0%). In detecting individuals with more subtle visual field perturbation (mean deviation of -2 dB or worse), sensitivity was 91.5% (95%CI: 86.4 to 94.8%) and specificity was 28.6% (95%CI: 18.4 to 41.5%), with a PPV of 80.1% (95 CI: 74.0 to 85.0%) and a NPV of 51.6% (95%CI: 34.8 to 68.0%).

### Participant feedback

**Fig 3:**
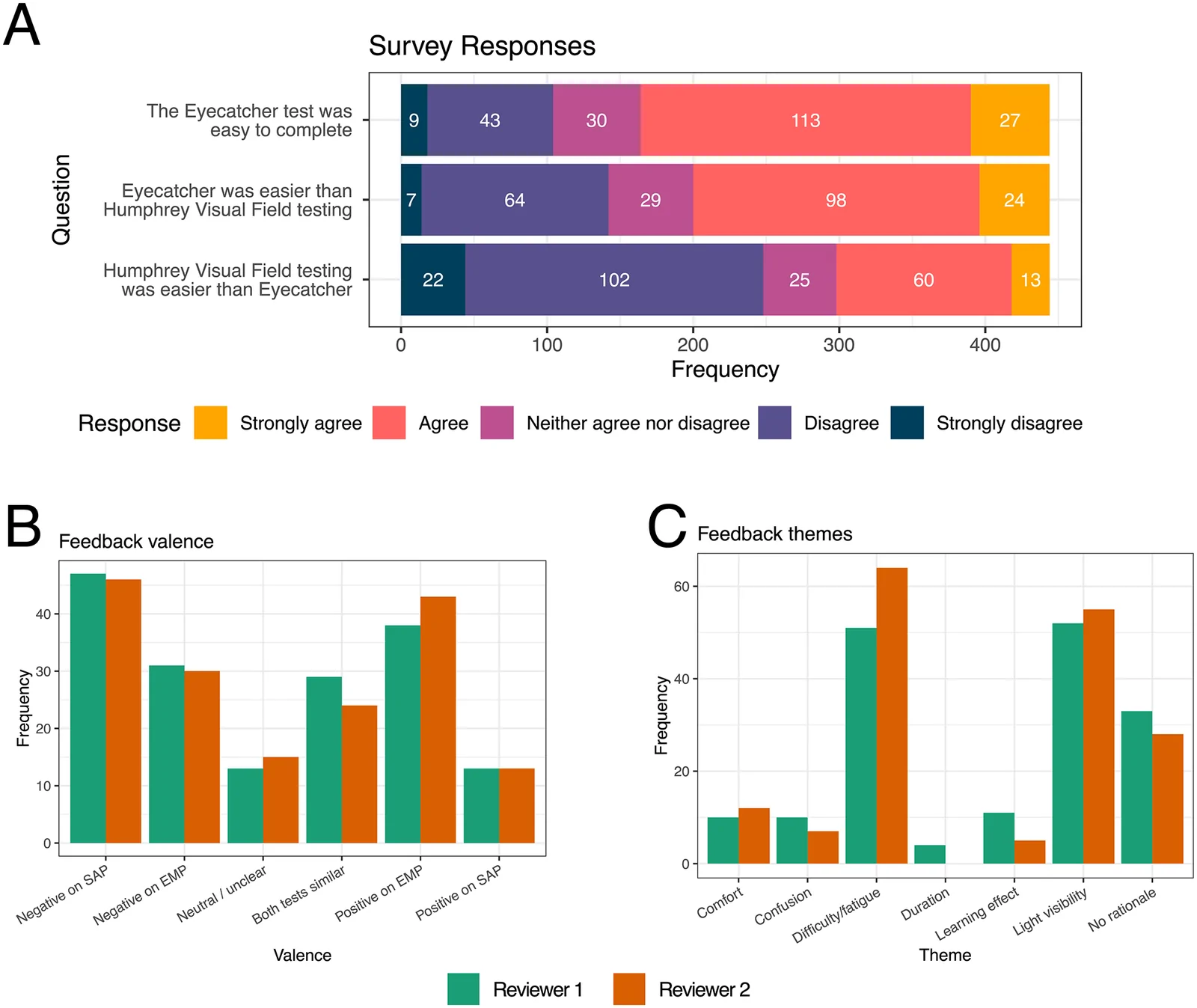
Feedback analysis from participants that undertook eye movement perimetry (EMP) and standard automated perimetry (SAP). The EMP test was known as ‘Eyecatcher’ during the study, reflected in the survey questions. SAP was referred to as ‘Humphrey Visual Field testing’. **A)** Relative frequency of participants reporting that the EMP test was easy to complete, that EMP was easier than SAP, and that SAP was easier than EMP. Most participants agreed that EMP was easy to complete and that EMP was easier than SAP. Most participants also disagreed that SAP was easier than the EMP test, showing consistency. **B)** Valence of participant feedback deduced through thematic analysis by two masked researchers. Negative comments were more frequent, but there were fewer negative comments concerning EMP than SAP, and more positive comments concerning EMP than SAP. **C)** Feedback themes deduced through thematic analysis. The most common themes identified were the difficulty or fatigue caused by perimetry tests and the visibility of light stimuli.

When surveyed, participants expressed favourable opinions about the EMP test (Fig 3A). 63.1% of participants agreed or strongly agreed that the EMP test was easy to complete. 55.0% of participants agreed or strongly agreed that EMP was easier than conventional SAP, and a similar proportion of 55.9% of participants disagreeing or strongly disagreeing that SAP was easier than EMP (the same question phrased in reverse). Thematic analysis of participants’ free text comments revealed six common concerns (Fig 3C): comfort (n = 10), confusion (n = 10), difficulty or fatiguability (n = 51), duration (n = 4), learning effects and novelty (n = 11), and light visibility (n = 52). In terms of valence, participants were more likely to provide negative comments than positive (Fig 3B). There were 31 negative comments about EMP and 47 negative comments about SAP; and 38 positive comments about EMP and 13 positive comments about SAP. 29 comments suggested both tests were similar, and 13 comments were neutral or unclear. Overall, participants were more positive and less negative about EMP than SAP.

## Discussion

In this study, EMP exhibited very good sensitivity in identifying 100% of individuals and eyes with visual field defects. Specificity was much lower (20.7% in person-level triage, 25.6% in eye-level analysis) due to many false positive cases as well as test failure. While a normal EMP test result provided strong reassurance of little or no visual field loss if tested with SAP, poor performance on the EMP test was inconclusive. In person-level triage emulation, while all patients with a severe defect on SAP were correctly flagged by EMP, up to four out of five patients with a poor EMP hit rate did not show a severe defect on SAP. Improving specificity is crucial to improve the utility of EMP for use as an initial triage tool to discharge individuals with no visual field defect [18]. This could reduce the number of individuals who require referral for formal SAP which is a crucial consideration in lower-income settings where equipment and operators are limited. Formal SAP may also require travel to a referral centre, incurring additional costs for patients. A considerable proportion of the false positive referrals were due to technical faults, showing where performance gains with little change to the test itself may be possible.

The participants’ comments suggest good acceptability of EMP as an alternative to SAP. This is consistent with an earlier pilot study of British glaucoma patients and healthy controls which also suggested that EMP is preferable as a test to SAP [15]. Other portable perimetry tests have similarly exhibited good acceptability and ease of use compared to SAP, ranging from screen-mounted eye-tracking to head-mounted or virtual reality systems [14,23–26]. The shorter duration and lower testing burden of EMP represent practical advantages that would support use as a tool for triaging patients with suspected glaucoma. These advantages stem from the incorporation of eye-tracking technology rather than requiring patients to fixate on a single point for many minutes, eye movements being the marker of sensitivity in the visual field, rather than relying on the sometimes stressful and frequently unreliable push-button response of SAP, as well as the lack of uncomfortable head fixation using a chin rest [27,28].

Test failure was frequent, limiting specificity and therefore the utility of EMP as a tool for triage. Failure was most frequently due to failure of initial calibration or issues with eye tracking. Various other factors may have contributed: the study was undertaken in real-world settings, in a busy ophthalmology outpatient department where patients were also being seen for their regular eye care visit and treatment; many patients were elderly, reflective of a glaucoma population; and patients in this Tanzanian clinic were likely less familiar with the technology used to complete the test. A previous study of the same test in a British cohort exhibited a much higher rate of completion, suggesting that familiarity with technology or differences in the testing environment may also have limited the EMP completion rate [18]. Moreover, differences between this study’s population and those with whom the eye tracking system has been developed—such as iris colour—may have contributed [29,30]. Emerging technology such as improved eye tracking, calibration-free spectacles-based devices, and virtual reality tests are anticipated to exhibit improved reliability and increased clinical potential [18,31]. A suite of portable tests incorporating EMP with visual acuity, tonometry, and imaging may also exhibit improved diagnostic accuracy as a whole [32,33].

This study was limited by its single centre design, which limited external generalisability. However it is plausible that findings would be similar in other sub-Saharan African or lower-income settings where potential glaucoma patients have similar exposure to mobile technology [34]. Recruiting one or both eyes from participants exposed the eye-level analyses of accuracy to within-subject clustering—this was done to enrich the data with glaucomatous eyes, and justified as glaucoma is frequently asymmetrical [35]. The person-level triage emulation was also undertaken as a more clinically and statistically faithful test of how EMP might be deployed if successful. No formal power calculation was undertaken as this was a feasibility study, although the narrowness of the confidence intervals for accuracy, sensitivity, and specificity indicate that the sample size was adequate for a preliminary assessment of EMP as an initial glaucoma test. An improved version of EMP should be tested in another prospective study with a prespecified analytical plan, powered to test whether performance meets required standards for wider use. The study would ideally be undertaken in more than one centre to maximise the generalisability of the findings.

This study showed that an EMP test has limited potential as a triage tool for initial visual field assessment. With its high sensitivity, EMP could help to reduce the number of individuals who require less accessible and more costly SAP to exclude a significant visual field defect. However, its poor specificity means that further work is required before it can be considered for clinical use. Technological developments are anticipated to lead to improved performance. Improving access to affordable, rapid, and more acceptable glaucoma diagnostics could help reduce the burden of irreversible vision loss by allowing more patients to access earlier treatment.

## Supporting information

S1 Text Prospectively registered protocol for the Glaucoma Detection Study (GDS) in Moshi, Tanzania. (PDF)

S1 Checklist ‘Inclusivity in global research’ checklist, mandated by PLOS. (DOCX)
