# Supplementary material for: Feasibility and performance of eye movement perimetry for glaucoma triage in Tanzania: A prospective cross-sectional study in a real-world setting
Source: PLOS Glob Public Health. 2026 Sep 25;6(9):e0007237. doi: 10.1371/journal.pgph.0007237 (PMC13614570; doi:10.1371/journal.pgph.0007237)
Supplement: Supplementary file 1 [file pgph.0007237.s001.pdf]

Glaucoma Detection Study, KCMC, Tanzania

Detecting glaucoma using low-cost, portable and easy to perform tests

Study Protocol
V 1.2
1st March 2020

Principal Investigator (KCMC, Tanzania): Einoti Matayan
Principal Investigator (LSHTM, UK): Victor Hu
Co-PI/Investigators: William Makupa, Karin Knoll, David Crabb, Matthew Burton, Winnie Nolan, Peter Jones, Heiko Philippin

Main Contacts

Principal Investigator in Tanzania, with overall local responsibility for the study:
Dr Einoti Matayan, Kilimanjaro Christian Medical Centre, Moshi, Tanzania
Email: blessednai23@gmail.com
Other local Co-PI and Co-Investigator who will support the PI:
Dr William Makupa, Kilimanjaro Christian Medical Centre, Moshi, Tanzania
Email: williamumakupa@yahoo.com
Dr Karin Knoll, Kilimanjaro Christian Medical Centre, Moshi, Tanzania
Email: karin.knoll@cbm.org
Chief Overseas Investigator:
Victor Hu, London School of Hygiene and Tropical Medicine, UK
Email: Victor.hu@lshtm.ac.uk
Tel. +44 7970 939 445

Other Co-investigators:
Professor David Crabb, City, University of London.
Email: David.Crabb.1@city.ac.uk
Professor Matthew Burton, London School of Hygiene and Tropical Medicine, UK
Email: Matthew.Burton@lshtm.ac.uk
Ms Winnie Nolan. London School of Hygiene and Tropical Medicine, UK.
Email: w.nolan@nhs.net
Dr Peter Jones, City, University of London.
Email: Peter.Jones@city.ac.uk
Dr Heiko Philippin, London School of Hygiene and Tropical Medicine, UK
Email: Heiko.Philippin@lshtm.ac.uk

Statistician: Min Kim, LSHTM, min.kim@lshtm.ac.uk

Local Research Assistant (patient recruitment, consent and study protocol):
Ms Sia Mbishi, Kilimanjaro Christian Medical Centre, Moshi, Tanzania
Email: SiaMbishi@gmail.com

For general queries, supply of trial documentation, and collection of data, please contact:
Victor Hu, as above.

Clinical Queries
Clinical queries should be directed to Victor Hu who will direct the query to the appropriate person.

Sponsor
London School of Hygiene & Tropical Medicine is the main research sponsor for this study. For further information regarding the sponsorship conditions, please contact the Research Governance and Integrity Office:
London School of Hygiene & Tropical Medicine
Keppel Street
London WC1E 7HT
Tel: +44 207 927 2626
Email:
LSHTM ethics reference: 17824

Funder
International Glaucoma Association
15 Highpoint Business Village, Henwood, Ashford, Kent, TN2 8DH

Tel. 01233 64 81 64
Email: r.kew@iga.org.uk

This protocol describes the Glaucoma Detection Study (GDS) and provides information about procedures for entering participants. The protocol should not be used as a guide for the treatment of other participants; every care was taken in its drafting, but corrections or amendments may be necessary. These will be circulated to investigators in the study, but centres entering participants for the first time are advised to contact the study centre to confirm they have the most recent version.

Problems relating to this study should be referred, in the first instance, to the study coordination centre.

This study will adhere to the principles outlined in the International Conference on Harmonisation Good Clinical Practice (ICH GCP) guidelines, protocol and all applicable local regulations.

Table of Contents

1 INTRODUCTION 6
1.1 SUMMARY 6
1.2 BACKGROUND 6
1.3 RATIONALE FOR CURRENT STUDY 8
2 STUDY OBJECTIVES 8
2.1 BROAD OBJECTIVE 8
2.2 SPECIFIC OBJECTIVES 8
3 METHODOLOGY 9
3.1 STUDY DESIGN 9
3.2 STUDY SITE 9
3.3 STUDY PARTICIPANTS 9
3.3.1 Inclusion Criteria 9
3.3.2 Exclusion Criteria 9
3.3.3 Withdrawal Criteria 9
3.4 PATIENT FLOW 9
3.5 DATA COLLECTION AND ANALYSIS 10
3.6 STUDY OUTCOME MEASURES 11
3.7 TIME FRAME 9
4 SAFETY REPORTING 12
4.1 DEFINITIONS 12
4.2 REPORTING PROCEDURES 12
4.2.1 Non serious AEs 12
4.2.2 Serious AEs 12
4.3 RISK ASSESSMENT 13
4.4 MONITORING AND DATA SAFETY 13
5 REGULARTORY CONSIDERATIONS 13
5.1 ETHICS APPROVAL 13
5.2 INFORMED CONSENT 13
5.3 CONFIDENTIALITY 13
5.4 SPONSOR 13
5.5 FUNDING 14
5.6 AUDITS AND INSPECTIONS 14
6 BUDGET 14
7 PUBLICATION POLICY 14
8 REFERENCES 14
9 APPENDICES 16
INVESTIGATION PROTOCOL
DATA COLLECTION FORM
SAE FORM

List of Abbreviations

AUC	Area Under the Curve
DDLS	Disc Damage Likelihood Scale
HVF	Humphrey Visual Field
GDS	Glaucoma Detection Study
KCMC	Kilimanjaro Christian Medical Center
KCMUCo	Kilimanjaro Christian Medical University College
NHS	National Health Service (UK)
NIMR	National Institute for Medical Research
ROC	Receiver Operating Characteristic
WHO	World Health Organization

Study Overview

TITLE	Detecting glaucoma using a combination of low-cost, portable, easy to perform tests: Glaucoma Detection Study (GDS)
DESIGN	Observational study.
AIMS	To develop a low-cost, portable glaucoma detection system which is easily performed and has good reliability and patient satisfaction.
OUTCOME MEASURES	Successful detection of glaucoma
POPULATION	Patients attending glaucoma outpatient clinics at Kilimanjaro Christian Medical Centre Eye Department, Tanzania
ELIGIBILITY	Attending a glaucoma outpatient clinic as above
DURATION	2 years for recruitment and follow-up

1 INTRODUCTION

1.1 SUMMARY GLAUCOMA DETECTION STUDY, KCMC, TANZANIA.

Glaucoma is a common cause of blindness and visual impairment worldwide and particularly in low-income areas such as sub-Saharan Africa. Damage to the optic nerve, usually associated with high pressure in the eye, leads to loss of the peripheral visual field and then loss of central vision and blindness. Detection and diagnosis of glaucoma is challenging and currently requires multiple tests using expensive equipment and highly trained personnel. Patients often present with advanced damage who have lost much of their visual function and who are at a very high risk of going completely blind. A detection system for glaucoma which is low-cost and can be easily performed by participants and operator would greatly help to detect cases of glaucoma at an earlier stage and reduce blindness.

The aim of this feasibility study is to develop a low-cost, portable glaucoma detection system which is easily performed and has good reliability and patient satisfaction. The study will be based at KCMC where around 200-300 participants will be recruited and a parallel but separate study will also be run at Mid Cheshire NHS Hospitals in the UK., recruiting around 120 participants. Participants will be recruited from patients attending ophthalmology outpatient appointments which encompasses a range from normal, healthy eyes to advanced glaucoma. A sample size of 320 participants allows a difference of 0.1 area under the Receiver Operating Curve to be detected and the confidence intervals to be determined.

Participants will undergo 3 tests which have all shown promise in detecting glaucoma and are also all quick and easy to perform and can be performed using equipment which costs a fraction of what is currently required for glaucoma testing. The three tests are:

1. Eyecatcher visual fields : have been developed which can be performed using a laptop screen and an eye-tracker which has shown good agreement with Humphrey visual fields. The Humphrey Field Analyser is the current method for visual field testing and costs over USD 20,000. Using the Eyecatcher visual fields, participants follow a series of dots on the laptop screen with their eyes. The use of the eyetracker means they do not have to keep their head fixed on a chin-rest.
2. PEEK Contrast Sensitivity: this is performed using a simple mobile phone. Participants indicate the orientation of letters on the screen. The letters get progressively dim until they cannot be seen anymore.
3. Optic nerve head photograph: this is performed using a portable camera, the Remidio camera. This has been CE marked and is FDA approved. It is in wide-spread clinical and research use and gives good quality photographs of the back of the eye.

The main objectives of the study are to see if the tests can be used to detect glaucoma and which combination of tests is best, to determine the repeatability of the tests, and also how participant feedback on how acceptable they find the tests. Data will be collected on data collection sheets and entered into a secure Access database. Regression analysis will be performed using Stata. Participant information will be kept strictly confidential. The main outcome measurement is if the glaucoma detection system can differentiate glaucoma patients versus healthy participants, and secondary outcomes include how well the Eyecatcher visual fields correlate with standard Humphrey fields.

The study will commence once ethical approval and insurance has been obtained, this is estimated to be June 2020 and will run until the end of 2021. Funding for this study has been obtained from the International Glaucoma Association. A budget of GB £20,500 has been allocated for this study which includes salary for a research assistant (£11,200), office expenses and local travel (£2000), ethics fees (£2500), patient costs (£3000) and contingency (£1800).

1.2 BACKGROUND

A recent systematic review found glaucoma to be the most common irreversible cause of blindness worldwide causing an estimated 2.9 million people to be blind and a further 4 million to have moderate or severe visual impairment.[1] It is the third most common cause of blindness overall, following cataract and uncorrected refractive error. The global prevalence of glaucoma for the population aged 40-80 years is estimated to be 3.5% with over 60 million people affected.[2] In the UK it is estimated that almost 10% in those older than 75% have chronic open angle glaucoma alone.[3] However, in low-income areas glaucoma prevalence appears to be much higher and it is a far more common cause of blindness. Recent population-based surveys from Sub-Saharan Africa found a prevalence of 4-8% in those over 40 years old with a high proportion of severe vision loss in both eyes.[1, 4-7] With an increasingly aging population the numbers of people with glaucoma across the world are expected to show large increases in the coming years.[7]

Despite its prevalence, diagnosing glaucoma is not straightforward, and confirming or excluding the presence of disease can be challenging. Even in developed countries up to half of glaucoma is undiagnosed, and this proportion is far lower in low-income settings.[7] A number of clinical tests and imaging modalities need to be performed, often sequentially over time.[3, 8] Our proposal is to assess and validate a combination of low-cost, portable, rapidly performed tests which can be brought together to function as a case detection system for glaucoma. The work funded by this grant will allow us to complete feasibility and reliability assessments for each of the tests and to optimise the glaucoma detection algorithm for maximum sensitivity and specificity.

Visual field examination is a central part of diagnosing and monitoring glaucoma. Standard automated perimetry is the current gold standard, usually performed with the Humphrey Field Analyser (HFA). However, Humphrey Visual Fields (HVFs) are time-consuming, difficult to perform and have a low patient satisfaction even in experienced test-takers, as well as requiring a trained operator to monitor the patient and ensure compliance.[9] The HFA machines are large and non-portable and are expensive to purchase and maintain, placing a financial burden on eye units and making them prohibitive in most low-income settings. This has led to an interest in developing alternatives to standard perimetry, including tablet or screen-based visual fields. Professor David Crabb's group (www.staff.city.ac.uk/crabblab), who have an impressive track record of research in visual field assessment and development, have developed a novel form of perimetry. "Eyecatcher" combines an inexpensive eye tracker with a simple portable tablet computer. Scores from Eye catcher demonstrates strong correlation with HVF mean deviation scores and good concordance between corresponding visual field locations.[10] The eye-tracking technology is used to monitor and compensate for fixation and patient movement during the test. This allows variation in the distance between the patient and the screen so that a chin-rest is not needed, adding to patient comfort. Participants reported that the Eyecatcher was more enjoyable, easier to perform and less tiring. A further study assessing the use of Eyecatcher fields in clinical practice is currently ongoing in the UK with preliminary experience confirming the positive results of the earlier laboratory-based study.

The Eyecatcher visual field test will form the first key component of the proposed glaucoma case detection system. The second test to be utilised will be digital photography of the optic disc. Optic disc assessment is critical to diagnosing and monitoring glaucoma and a high-quality colour photograph of the disc will help to determine if glaucomatous damage has occurred.[3, 8] Retinal and optic disc imaging is a rapidly developing field and technological advancements are helping to drive down costs, a pattern which is likely to continue in the future. This study will use the Remidio hand-held fundus camera which is able to take high quality images with the use of a smart-phone.[11] We have had good experience of this camera with diabetic retinopathy studies in Zamiba. This study will use expert graders to grade the optic discs for glaucomatous damage, however, we anticipate that in the future machine learning will help to automate this process. Artificial intelligence based automated software for detecting diabetic retinopathy from retinal images has reached an advanced stage and similar work has begun on glaucoma detection.[11-13]

The third test to be evaluated is contrast sensitivity. There is good evidence that contrast sensitivity is able to differentiate between glaucomatous and healthy eyes and should be used much more in standard clinical practice.[14-17] PEEK contrast sensitivity is a newly developed test, which can be performed on a smart phone or tablet computer, is a repeatable and rapid test and is highly comparable to Pelli-Robson contrast sensitivity.[18]

Each of the three proposed tests are rapid, easily performed and require relatively low-cost equipment (especially considering future advances) with a portable set-up. They can be performed by allied health care workers without the need for extensive training. A single electronic device, such as a tablet computer, can currently be used to perform two of the tests, the Eyecatcher fields and contrast sensitivity. Recent technological advancements strongly point to such a device also being able to obtain high quality optic disc images in the future. This device can be used to securely store all of the data collected, including relevant patient demographic parameters, and could also potentially contain software helping to interpret and analyse this data. We also plan to retrospectively gather clinical data from ophthalmology patients which is of relevance. A similar study will be done in England and we also plan to combine or compare results with this study

This study would form an important part of a wider body of glaucoma research being undertaken by the London School of Hygiene and Tropical Medicine. A key aim of this work is to promote the development of a low-cost system for glaucoma detection for use in communities that are poorly resourced in areas difficult to reach. Equipment and a diagnostic protocol will be developed that can be used by non-medical personnel. The ongoing research work will be able to test the glaucoma detection system developed in this study and validate its usefulness in ongoing studies in the community. Our long-term aim is for a single device to be used to perform all the clinical tests, analyse the data according to an optimised algorithm, and give a likelihood ratio score for the presence of glaucoma. This study is part of the Kilimanjaro Glaucoma Innovation Programme (KiGIP).

1.3 RATIONALE FOR CURRENT STUDY

This study has the potential to make great progress in glaucoma detection. Up to half of glaucoma in high resource setting is undiagnosed, and this proportion is far higher in low resource setting. Glaucoma is the most common irreversible cause of blindness worldwide and this study aims to help reduce this burden of blindness.

Detecting and diagnosing glaucoma is challenging, often requiring expensive, complex equipment and repeated examinations. This study is a feasibility study of a number of simple, non-invasive tests to see if they can be used for detecting glaucoma.

2 STUDY OBJECTIVES

2.1 BROAD OBJECTIVE

To detect glaucoma using low-cost, portable and easy to perform tests. We will do this by testing the feasibility and reliability of a number of diagnostic tests which can be combined to form a novel glaucoma case detection system.

2.2 SPECIFIC OBJECTIVES

- To determine whether the 3 proposed tests (Eyecatcher visual fields; PEEK contrast sensitivity; optic disc photography) can be used as a case detection system for glaucoma.
- To measure the repeatability of each of the tests and see if this acceptable.
- To determine the utility of each test to differentiate glaucomatous damage from normal, and the optimum combination of tests for this purpose.
- To measure the patient acceptability of each test using a Usability Questionnaire.
- To describe routine exam results and other characteristics, also retrospectively, of patients who attended the eye clinic mainly to provide a comparison for the results from this study

- To compare the results with findings from similar studies, e.g. the twin study in the UK as part of the same Glaucoma Detection Study funded by IGA

3 METHODOLOGY

3.1 STUDY DESIGN

This is a cross-sectional diagnostics study.

3.2 STUDY SITE

Participants will be recruited from Kilimanjaro Christian Medical Centre (KCMC), Tanzania.

3.3 STUDY PARTICIPANTS

Participants will be recruited from patients who are attending for ophthalmology appointments in the outpatient departments. This is a heterogenous population and will allow us to include patients ranging from those with established glaucoma and clear visual field defects, early glaucoma, glaucoma suspects, ocular hypertension and also healthy controls (for example, new referrals with no disease). A similar recruitment protocol has been used in the current Eyecatcher visual field study which has successfully recruited 80 participants to date.[19]

3.3.1 Inclusion Criteria

- Age ≥ 18 years.
- Giving informed consent;
- The ability to understand and cooperate with the tests.

3.3.2 Exclusion Criteria

- Not meeting the inclusions criteria.
- Previous participation in the study.

3.3.3 Withdrawal Criteria

Participants can withdraw consent at any stage. If they also wish their data to be destroyed, then it will be destroyed accordingly.

3.4 STUDY TESTS AND PATIENT FLOW

3.4.1 Patient Flow

While waiting to be seen patients will be invited to the join the study and, if willing, informed consent for enrolment will be obtained. A routine examination is performed and patients will receive a treatment recommendation as usual. Standard OCT and visual field diagnostics will be performed as well. For the purpose of the study, participants will complete the following tests described below.

We anticipate that participants will spend around 30-45 minutes with the research study, including recruitment, giving informed consent and completing the study tests, including repeating the tests if the participant is happy to do so. We would ensure that this involves no, or minimal, disruption to their regular clinic visit. Patients in the glaucoma clinic often spend time waiting to be seen and we would aim to use this time for the research study. If necessary, the research study protocol could be completed after the regular clinic, but only if the participant is happy with this.

3.4.2 Study Tests and Feedback

The study protocol will involve the three tests below:

1. Eyecatcher visual fields: the protocol for this is well established and has shown good correlation with HVF results, as above. A tablet computer or laptop screen can be used, with a clip-on eye tracker such as the Tobii EyeX. The diagnostic test is done similar as follows: a quick, screening field test is done with supra-threshold light intensity, taking 1-2 minutes. Participants who are able to perform this initial test will go on to be tested with light intensity stimuli equivalent to ~10 dB on the HFA. The subsequent analysis allows pointwise concordance with HFA locations to be computed with an equivalent sensitivity, for example, of 25 dB. This will take around 5 minutes, but the patient experience compared to HVF is improved as the eye-tracking technology can compensate for patient movement and fixation. Patient acceptability will be measured. Participants will be asked if they are willing to repeat the Eyecatcher fields before leaving the department so that we can assess the retest variability. The Eyecatcher field analysis will be graded as indicating glaucomatous damage or no damage
2. Contrast sensitivity: Peek Contrast Sensitivity will be measured on a handheld device. The screen presents a letter "E" in random orientations and the subject indicates the correct orientation. The test starts with maximal contrast which then decreases until the subject's contrast detection threshold is determined. A logarithm score of between 0.00 and 2.25 log units is generated. The test takes less than a minute complete. Other tests of the Peek platform, e.g. the visual acuity test will be performed as well.
3. Optic disc photo: the Remidio hand-held fundus (or comparable) camera is able to take good quality optic disc images without pupil dilation in a dim room or with the use of a small gown. Image acquisition takes around 2-3 minutes. The camera has a well-developed software package which allows easy extraction and transfer of images. Images will be graded by experts e.g. using the disc damage likelihood scale and graded as indicating glaucomatous change or not.[20-22]

Participant feedback on the tests will be collected using the Usability Questionnaire. Participants will be asked the questions and the researcher will complete this form.

3.5 DATA COLLECTION AND ANALYSIS

Data will be collected according to the data collection sheet (see attachment) and from the patient file including (among others):

- Age, sex and ethnicity
- Diagnosis and co-morbidity
- Visual acuity
- Intraocular pressure
- Central corneal thickness
- HVF analysis (preferably same-day, or within a 3-month period)

Data which will be collected by the research protocol will be as follows:

1. Eyecatcher visual fields: Participants tested with light intensity stimuli equivalent to ~10 dB on the Humphrey Field anaylser. The subsequent analysis allows pointwise concordance with HFA locations to be computed with an equivalent sensitivity, for example, of 25 dB. The Eyecatcher field analysis will be graded as indicating glaucomatous damage or no damage.
2. Contrast sensitivity: Peek Contrast Sensitivity will be measured on a smart phone. The tests starts with maximal contrast which then decreases until the subject's contrast detection threshold is determined. A logarithm score of between 0.00 and 2.25 log units is generated.
3. Optic disc photo: the Remidio camera images will be uploaded to a secure HIPAA (Health Insurance Portability and Accountability Act) compliant cloud server. Images will be graded by experts using the disc damage likelihood scale and graded as indicating glaucomatous change or not.

A formal sample size calculation is challenging at this stage as this is a feasibility study of a novel system. However, a sample of 146 from the positive group (cases) and 146 from the negative group

(controls) achieves 85% power to detect a difference of 0.10 between a diagnostic test with an area under the Receiver Operating Curve (ROC) curve (AUC) of 0.60 and another diagnostic test with an AUC of 0.70 using a two-sided z-test at a significance level of 0.05. The data are continuous responses with the AUC computed between false positive rates of 0 and 1. The correlation between the two diagnostic tests is assumed to be r=0.5 for the positive group and r=0.5 for the negative group [1,2,3]

Using a sample size of 320 with n=160 cases allows an estimate of the width of the confidence interval around the estimates of sensitivity. For example, if a test has a sample sensitivity of 60% then the confidence interval (CI for one proportion from the binomial distribution) will be 52-68%. These sensitivity values for the different instruments will be relevant at a fixed specificity: 90% specificity will be reasonable and the sample size of 160 controls is sufficient to give a 95% CI around this estimate of 84-94%. These limits will be published with all our results following recommendation from [4].

[1] https://www.ncbi.nlm.nih.gov/pubmed/6878708
[2]Obuchowski, N. and McClish, D. (1997) Sample Size Determination for Diagnostic Accuracy Studies Involving Binormal ROC Curve Indices. Statistics in Medicine 16: 1529-42.
[3] https://www.ncss.com/software/pass/
[4] https://www.ncbi.nlm.nih.gov/pmc/articles/PMC1955666/

The grant size, timeline and logistics allow recruitment of 200-300 participants at KCMC which is consistent with the 320 calculated from the above sample size calculation once the additional participants from the UK (120) are included.

Any participants' identifiable data collected will be stored securely and confidentiality strictly maintained. Data and all appropriate documentation will be stored for a minimum of 5 years after the completion of the study, including the follow-up period.

Our intention is to recruit participants with a wide range of pathology and glaucoma severity, from healthy eyes to end-stage disease. We will also retrospectively access and collect relevant clinical data of ophthalmology patients who are not being included in the prospective study above. This data would be similar to that collected from study participants:

- Age, sex and ethnicity
- Diagnosis and co-morbidity
- Visual acuity
- Intraocular pressure
- Ocular examination results
- Central corneal thickness
- HVF analysis (preferably same-day, or within a 3-month period)

3.6 STUDY OUTCOME MEASURES

Primary outcome measure of the glaucoma detection system will be established diagnosis of glaucoma *versus* healthy control. We want to establish whether our proposed system can differentiate those who have a diagnosis of glaucoma from those with no disease in comparison to the respective gold standard diagnostic devices. Regression analysis will be performed for the different tests, and combination of tests, to optimise the receiver operating characteristic (ROC) for the area under the curve (AUC) for glaucoma detection.

Secondary outcomes include mean deviation on HVF testing: this is well-established means of assessing glaucoma severity and we will measure how well this can be predicted by the case detection system. Furthermore, the detection of differential diagnoses (in particular cataract) will be evaluated as well as other descriptors of the disease of glaucoma.

Data analysis will take place at the London School of Hygiene and Tropical Medicine using anonymised data only.

3.7 TIME FRAME

Recruitment will commence once ethical approval and insurance has been obtained, this is estimated to be June 2020 and will run until the end of 2021. Analysis will be performed at the start of 2022 with publication and dissemination of results planned for later that year.

4 SAFETY REPORTING

4.1 DEFINITIONS

Term	Definition
Adverse Event (AE)	Any untoward medical occurrence in a patient or study participant
Serious Adverse Event (SAE)	A serious event is any untoward medical occurrence that:<br>Results in death<br>Is life-threatening<br>Requires inpatient hospitalisation or prolongation of existing hospitalisation<br>Results in persistent or significant disability/incapacity<br>Consists of a congenital anomaly or birth defect<br><br>Other ‘important medical events’ may also be considered serious if they jeopardise the participant or require an intervention to prevent one of the above consequences.

4.2 REPORTING PROCEDURES

Depending on the nature of the event the reporting procedures below should be followed. Any questions concerning adverse event reporting should be directed to the Chief Investigator in the first instance.

4.2.1 Non serious AEs

Any adverse events will be recorded in the patient notes and in the Adverse Event log.

4.2.2 Serious AEs

Serious Adverse Events (SAEs) should be reported to the study coordination centre within 24 hours of the local site being made aware of the event.

An SAE form should be completed and submitted to the study coordination centre with as much detail of the event that is available at that time. If awaiting further details, a follow up SAE report should be submitted promptly upon receipt of any outstanding information.

Any events relating to a pre-existing condition or any planned hospitalisations for elective treatment of a pre-existing condition do not need reporting as SAEs.

Contact details for reporting SAEs
Please send SAE forms to:
Dr. Victor Hu
Email: victor.hu@lshtm.ac.uk
Tel: +44 7970 939 445 (Mon to Fri 09.00 – 17.00)

4.3 RISK ASSESSMENT

This study is a non-interventional study using three tests which are non-interventional with low/negligible risk.
All of the test are extremely low risk, non-invasive procedure and non-contact. The Eyecatcher visual fields uses eye-tracker with non-harmful infrared light to track the eyes. The contrast sensitivity uses a carefully calibrated light source which becomes increasingly dim. The optic disc photography uses a non-harmful light to illuminate the optic disc for the photo.
The main concern is that the patient may become frustrated or bored with the tests. If this appears to be the case then researchers are able to suspend the test and allow the patient to take a break. Furthermore, the tests are designed to be as short, comfortable, and engaging as possible, specifically to minimise the risk of participants becoming bored.

4.4 MONITORING AND DATA SAFETY

Data entry and consent form checks will be made by the CI and co-investigators. Data entry will be made in real time into a laptop, with regular data back up to secure external hard drives. Data will be analysed for outlying values and these will be verified.
This data will help to inform the main, prospective part of the study. Patient identifiable data would not be collected for this, only the patient hospital number for reference. Data will be input into a password protected, encrypted laptop at the hospital sites, and the laptop stored securely. Transfer of data from this laptop will involve removal of the hospital number so that data is completely de-identified.

5 REGULARTORY CONSIDERATIONS

5.1 ETHICS APPROVAL

Approval has been obtained from the LSHTM Research Ethics Committee on 29th October 2019.
Approval will also be applied for from the National Institute for Medical Research in Tanzania.

Substantial amendments will not be implemented until favourable ethical approval has been gained.
Progress reports will be made as appropriate to the various ethics committees and all procedures and requirements will be adhered to as appropriate.

5.2 INFORMED CONSENT

Informed consent must be obtained prior to the participant undergoing procedures that are specifically for the purposes of the study.
Consent to enter the study must be sought from each participant only after a full explanation has been given, an information leaflet offered and time allowed for consideration. Signed participant consent should be obtained.
The right of the participant to refuse to participate without giving reasons must be respected.
All participants are free to withdraw at any time from the protocol treatment without giving reasons and without prejudicing further treatment.
The PI is responsible for ensuring that all vulnerable participants are protected and participate voluntarily in an environment free from coercion or undue influence.

5.3 CONFIDENTIALITY

Any participants' identifiable data collected by the Study Coordination Centre will be stored securely and their confidentiality protected in accordance with the Data Protection Act 1998.
Participant confidentiality will be strictly maintained. Data will be stored using identification numbers with no identifying information. General Data Protection Regulation will be adhered to.

5.4 SPONSOR

London School of Hygiene & Tropical Medicine will act as the main sponsor for this study. Delegated responsibilities will be assigned locally.

5.5 FUNDING

The International Glaucoma Association are funding this study.

5.6 AUDITS AND INSPECTIONS

The study may be subject audit by the London School of Hygiene & Tropical Medicine under their remit as sponsor, the Study Coordination Centre and other regulatory bodies to ensure adherence to GCP.

6 BUDGET

		Budget (£)	Narrative Budget Justification
1	Research assistant	£11,200.00	An experienced research assistant will be employed full-time for 24 months and additional support will be recruited when necessary
2	Office expenses & local travel	£2,000.00	Miscellaneous office expenses of the KCMC Eye Health / KiGIP Office such as but not limited to stationary, photocopying, local travel within Tanzania during the period of the study, e.g. to travel to the bank to deposit fees for ethical clearance or to buy stationary.
3	Ethics fees (KCMUCo and NIMR)	£2,500.00	Submission fees and annual extensions of ethical approvals at KCMUCo and NIMR. It includes the international rates for expedited clearance at NIMR for a Health Research Proposal of 1000 US$, renewal of approval at NIMR for year 2 of 100 US$. Furthermore fees at KCMUCo of approval of 500 US$ and annual extension fee of 100 US$. In case of changes in the respective fee structures, a contingency is included which can be used for other budget lines if it is not needed.
4	Patient cost (diagnostic fees, etc.)	£3,000.00	Refund for patients attending KCMC eye department of diagnostic fees e.g. Humphrey visual field examination and other patient related cost
5	Contingency	£1,800.00	Contingency for change of costing of the above, regular maintenance of equipment or replacement of broken equipment such as light bulbs, tonometer heads etc.
	**Total**	**£20,500.00**	

7 PUBLICATION POLICY

Results will be published in a peer-reviewed journal. All individuals that have had significant input to the study will be listed as co-authors or acknowledged, as appropriate.

8 REFERENCES

1. Flaxman SR, Bourne RRA, Resnikoff S, et al. Global causes of blindness and distance vision impairment 1990-2020: a systematic review and meta-analysis. *Lancet Glob Health* 2017;5:e1221-e1234.
2. Tham YC, Li X, Wong TY, Quigley HA, Aung T, Cheng CY. Global prevalence of glaucoma and projections of glaucoma burden through 2040: a systematic review and meta-analysis. *Ophthalmology* 2014;121:2081-2090.
3. Glaucoma: diagnosis and management. National Institute of Health and Clinical Excellence, UK. 2017.
4. Budenz DL, Barton K, Whiteside-de Vos J, et al. Prevalence of glaucoma in an urban West African population: the Tema Eye Survey. *JAMA Ophthalmol* 2013;131:651-658.

5. Kyari F, Abdull MM, Bastawrous A, Gilbert CE, Faal H. Epidemiology of glaucoma in sub-saharan Africa: prevalence, incidence and risk factors. *Middle East Afr J Ophthalmol* 2013;20:111-125.
6. Preussner PR, Grossmann A, Ngounou F, Kouogan G, Tamon J. Glaucoma screening in Western Cameroon. *Graefes Arch Clin Exp Ophthalmol* 2009;247:1671-1675.
7. Quigley HA. Glaucoma. *Lancet* 2011;377:1367-1377.
8. Primary Open Angle Glaucoma Preferred Practice Pattern. American Academy of Ophthalmology. 2015.
9. Glen FC, Baker H, Crabb DP. A qualitative investigation into patients' views on visual field testing for glaucoma monitoring. *BMJ Open* 2014;4:e003996.
10. Jones PR, Smith ND, Bi W, Crabb DP. Portable Perimetry Using Eye-Tracking on a Tablet Computer-A Feasibility Assessment. *Transl Vis Sci Technol* 2019;8:17.
11. Rajalakshmi R, Subashini R, Anjana RM, Mohan V. Automated diabetic retinopathy detection in smartphone-based fundus photography using artificial intelligence. *Eye (Lond)* 2018;32:1138-1144.
12. Kim SJ, Cho KJ, Oh S. Development of machine learning models for diagnosis of glaucoma. *PLoS One* 2017;12:e0177726.
13. Du XL, Li WB, Hu BJ. Application of artificial intelligence in ophthalmology. *Int J Ophthalmol* 2018;11:1555-1561.
14. Richman J, Lorenzana LL, Lankaranian D, et al. Importance of visual acuity and contrast sensitivity in patients with glaucoma. *Arch Ophthalmol* 2010;128:1576-1582.
15. Breton ME, Wilson TW, Wilson R, Spaeth GL, Krupin T. Temporal contrast sensitivity loss in primary open-angle glaucoma and glaucoma suspects. *Invest Ophthalmol Vis Sci* 1991;32:2931-2941.
16. Richman J, Zangalli C, Lu L, Wizov SS, Spaeth E, Spaeth GL. The Spaeth/Richman contrast sensitivity test (SPARCS): design, reproducibility and ability to identify patients with glaucoma. *Br J Ophthalmol* 2015;99:16-20.
17. Klein J, Pierscionek BK, Lauritzen J, Derntl K, Grzybowski A, Zlatkova MB. The Effect of Cataract on Early Stage Glaucoma Detection Using Spatial and Temporal Contrast Sensitivity Tests. *PLoS One* 2015;10:e0128681.
18. Habtamu E, Bastawrous A, Bolster N, et al. Development and validation of a smartphone-based contrast sensitivity test. *Ophthalmology* 2019;Submitted.
19. IRAS #230440: Eyecatcher Lite: Feasibility assessment of a tablet-based eye-tracking platform to screen for visual field loss.
20. Spaeth GL, Lopes JF, Junk AK, Grigorian AP, Henderer J. Systems for staging the amount of optic nerve damage in glaucoma: a critical review and new material. *Surv Ophthalmol* 2006;51:293-315.
21. Kara-Jose AC, Melo LAS, Jr., Esporcatte BLB, Endo A, Leite MT, Tavares IM. The disc damage likelihood scale: Diagnostic accuracy and correlations with cup-to-disc ratio, structural tests and standard automated perimetry. *PLoS One* 2017;12:e0181428.
22. Spaeth GL, Henderer J, Liu C, et al. The disc damage likelihood scale: reproducibility of a new method of estimating the amount of optic nerve damage caused by glaucoma. *Trans Am Ophthalmol Soc* 2002;100:181-185; discussion 185-186.
23. Foster PJ, Buhrmann R, Quigley HA, Johnson GJ. The definition and classification of glaucoma in prevalence surveys. *Br J Ophthalmol* 2002;86:238-242.

APPENDICES

Appendix A: Investigation Protocol

1. Consent and Information

Informed Consent is the process by which a potential study participant voluntarily confirms his or her willingness to participate in a study, having been informed of the full details of the project. Informed consent is normally documented by means of a written, signed and dated informed consent form.

Informed consent is a three step process which involves:
1. Giving of information
2. Discussion and clarification of the given information
3. Taking the participant's oral consent, with written consent where feasible

The person seeking informed consent will be a research study assistant according to ICH GCP section 4.8. (1996) and Declaration of Helsinki, section 26 (1964). The eligible patient is asked if he prefers information in English or Swahili and the information sheet is handed out accordingly. If the patient is not able to read, the accompanying helper or the person seeking informed consent reads it to the patient. Possible participants will be informed about the nature of the study and asked if they would be willing to participate, to be randomised to alternative treatment arms and to be followed-up for one year.

The staff seeking consent confirms that the patient has read or was read the information sheet and asks the patient if he has any more questions, concerns or suggestions. All issues are discussed with the potential study participant.

All parts of the consent form are read to the patient and the boxes ticked accordingly. The patient is asked to sign or provide a thumb print on two consent forms (one for the patient, one for the records). In case the patient is unable to read, an impartial witness needs to confirm with his signature that all the information about the study was given and the participant consented to taking part. The completed consent forms will be scanned, filed and stored in the project office.

2. Background information section

- The paper data collection form will be completed (see below).
- ID for pilot participants: GDS001, GDS002, etc
- This information will be collected by the research study assistant.

23 PEEK Contrast sensitivity

- CS should be measured using the Sony Experia phone according to the application guidelines.
- Patient should seated be 1 metre from the phone in dim lighting.
- Occlude other eye while measurement taken.
- The contrast is progressively reduced until the patient cannot see the letter displayed.
- The test will be performed by the research study assistant.

4. Eyecatcher visual field

- Patient is next moved to face the Surface Pro laptop for the Eyecatcher VF:
- Laptop should be attached to the power cable when being used, and brightness kept on maximum.
- Adjust the height of the laptop so that the patient is comfortably facing the screen, check the patient's eyes are 55cm from the screen at the beginning of the test (the patient only needs to remain reasonably still during the test, it is not necessary to recheck the distance as the eyetracker can adjust for small movements).
- If it takes a while for the patient to understand the test and follow the instructions then press Esc and restart the test once they can follow the instructions. The test can be restarted a further time if necessary, but not more than twice.
- The test will be performed by the research study assistant.

5. Remidio optic disc photos

- Use the GDS study number in the patient details section.
- Participant places chin on the rest.
- Keep the settings on the default - infrared light to visualise the optic disc and white light to take the photo (warn patient there will be a bright flash).
- Camera is gently brought forward until the optic disc is well centred and the image is as bright all the way around (or as much as possible).
- Press the image of the optic disc on the screen so that this area is focused on and take the photo.
- 3 photos of each eye to be taken.
- (If the pupils are very small it may not be possible to obtain good quality images).
- The test will be performed by the research study assistant.

6. Patient acceptability questionnaire

- Complete the questions and the comments.
- The test will be performed by the research study assistant.

Supervision

Supervision of the research study assistant will be performed by the Principal Investigator, Dr. Einoti Matayan, or delegated to the co-investigators.

Appendix B: Data collection form

1. Background information

GDS Study number	TestGDS001…	
Examiner	Sia<br>Edith	▢<br>▢
Date of examination	Date / Month / Year	
First name		
Last name		
Date of birth	Date / Month / Year	
KCMC number		
Ethnic group		
Sex	Male<br>Female	▢<br>▢

2. GDS Data

	Pin hole	**R**	**L**
	Glasses	**R**	**L**
PEEK contrast sensitivity		**R**	**L**
PEEK contrast brightness		**R**	**L**
Eyecatcher VF completed	Yes<br>No	▢<br>▢ **R**	▢<br>▢ **L**
Reason if Eyecatcher VF not completed	Eye tracker not able to track the eyes<br>Patient unable to follow instructions<br>Other (specify)	▢<br>▢<br>▢ **R**	▢<br>▢<br>▢ **L**
Remidio disc photo done?	(3 photos to be done)<br>Yes<br>No	▢<br>▢ **R**	▢<br>▢ **L**

3. Patient feedback on Eyecatcher VFs

Eyecatcher VF was easy to perform	Strongly disagree<br>Disagree<br>Neither agree or disagree<br>Agree<br>Strongly agree	▢<br>▢<br>▢<br>▢<br>▢
Eyecatcher VF were easier to perform than HVFs	Strongly disagree<br>Disagree<br>Neither agree or disagree<br>Agree<br>Strongly agree	▢<br>▢<br>▢<br>▢<br>▢
Comments		

Appendix C: Serious Adverse Event Reporting Form

SERIOUS ADVERSE EVENT REPORTING FORM

TITLE OF TRIAL: GLAUCOMA DETECTION STUDY

Please send to victor.hu@lshtm.ac.uk within 24 hours of notification of event

Patient Initials: ……………………………………………………….…	Patient Study No: ☐☐☐☐☐☐☐☐
Treating Clinician: …………………………………………………….…	Hospital/Site: …………………………………………………………..….

Type of Report

☐ 1=First
2=Interim
3=Final

Age at time of event ☐☐☐

.1 Sex

☐ 1= Male
2= Female

8.1.2
8.1.3
8.1.4

Why was the event serious? *(choose most serious)*

☐ 1= Resulted in death
2= Life-threatening
3= Required inpatient hospitalisation or prolongation of existing hospitalisation
4= Resulted in persistent or significant disability/incapacity
5= Resulted in congenital anomaly/birth defect
6= Other medically important event

Where did the SAE take place?

☐ 1= Hospital
2= Out-patient clinic
3= Home
4= Nursing home
5= Hospice
6= Other, specify…………………………………………….

Briefly describe SAE (*include relevant symptoms, body site, and relevant lab tests, treatments received)*
continue on a separate sheet if necessary

8.1.5 Details of SAE			
Serious Adverse Event Name:	8.1.6 Duration of SAE (dd mmm yy)	8.1.7 SAE Status<br>1= Resolved<br>2= Resolved with sequelae<br>3= Persisting<br>4= Worsened<br>5= Fatal<br>6= Not assessable	8.1.7.1.1.1 Expectedness<br>1= Expected*<br>2= Unexpected
8.1.8 Name<br><br>**Grade** ☐	Date of Onset<br>☐☐ ☐☐☐ ☐☐<br>Date Resolved<br>☐☐ ☐☐☐ ☐☐<br>or tick box if ongoing ☐	☐	☐

* *Was the event one of the recognised undesirable effects of the trial medication? See page xx of protocol*

8.1.9 Other relevant information to facilitate assessment
(Include medical history, drug or alcohol abuse, family history, findings from special investigations)

8.1.10 Was this event expected in view of the patient's clinical history?

☐ 0= No
1= Yes

Additional Information:

8.1.11 Signature

8.1.11.1.1.1.1 Authorised Health Professional ....

8.1.12 Print name..

8.1.13 Contact telephone no..

Date of report ☐☐ ☐☐ ☐☐
d d m m y y

FOR OFFICE USE ONLY

Person/Organisation received by (Please print): ______________________________

Signature:_____________________ Date: ☐☐ ☐☐ ☐☐
d d m m y y

Comments:
____
____
____

Confirmation of event in medical terms:
____

PV review – to be completed if a SAR is being reviewed:

Is the event expected? ☐ Y ☐ N

If expected, state what AR it is on the RSI: ___________________ RSI version:

Expedited reporting required? ☐ Y ☐ N

If yes, date completed: ☐☐ ☐☐ ☐☐
d d m m y y

Name of clinical reviewer (please print): ______________________________

Signature: ______________________________ Date: ☐☐ ☐☐ ☐☐
d d m m y y

FOR COMPLETION AFTER SUBMISSION OF REPORTS TO EC and RA

Date reported to LSHTM REC: ☐☐ ☐☐ ☐☐ N/A
d d m m y y

Date of receipt/acknowledgement: ☐☐ ☐☐ ☐☐
d d m m y y

Date reported to Local REC: ☐☐ ☐☐ ☐☐ ☐ N/A
d d m m y y

Date of receipt/acknowledgement: ☐☐ ☐☐ ☐☐
d d m m y y

Date reported to Regulatory Authority: ☐☐ ☐☐ ☐☐ ☐ N/A
d d m m y y

Date of receipt/acknowledgement: ☐☐ ☐☐ ☐☐
d d m m y y
